# Evaluation of Fracture Toughness, Color Match, and Handling of Resin Composite Restorations with Different Dentin-Replacement Materials

**DOI:** 10.3390/polym18141754

**Published:** 2026-07-17

**Authors:** Maryam A. Alghilan, Norah K. Alshammari, Fay A. Alammar, Mozoon N. Almohaiza, Muhammad I. Khan

**Affiliations:** 1 Department of Restorative and Prosthetic Dental Sciences, College of Dentistry, King Abdullah International Medical Research Center, King Saud bin Abdulazis University for Health Sciences, Ministry of National Guard Health Affairs, Riyadh 11481, Saudi Arabia; 2 College of Dentistry, King Saud bin Abdulazis University for Health Sciences, Riyadh 11481, Saudi Arabia; alshamari167@ksau-hs.edu.sa (N.K.A.); alammar147@ksau-hs.edu.sa (F.A.A.); almohaiza159@ksau-hs.edu.sa (M.N.A.); 3 Division of Biostatistics, Department of Population Health, King Abdullah International Medical Research Center, King Saud bin Abdulaziz University for Health Sciences, Ministry of National Guard Health Affairs, Riyadh 11481, Saudi Arabia

**Keywords:** bulk-fill composites, short fiber-reinforced composites, resin-modified glass ionomers, fracture toughness, color match, application efficiency, biomimetic dentistry, dentin-replacement materials

## Abstract

Evaluation of dentin-replacement materials (DRMs) is essential for optimizing material selection and restorative outcomes. Four restorative groups, each comprising a DRM (SDR^®^ Plus, group A; EverX Posterior^™^, group B-control; Filtek^™^ Z250, group C; Fuji II LC^®^, group D) overlayed with a microhybrid composite (Filtek Z250) to replace enamel layer, were evaluated for mechanical, optical, and handling characteristics. Standardized specimens were prepared for color change (ΔEab/E00) and fracture toughness (K_IC_) testing (n = 8/group/test), with application time and handling evaluated by two independent assessors. Data were collected and analyzed statistically. The greatest color difference was observed in group B, which was significantly higher than that in groups A and C (*p* < 0.05), while group D did not differ significantly from any other group. Groups A and B exhibited significantly higher (*p* < 0.001) fracture toughness than groups C and D with no significant differences within either pair of groups. Group A required the least application time (*p* < 0.001), followed sequentially by groups B, C, and D. Handling ratings varied by material, with moderate inter-rater reliability (κ = 0.53). Within the study’s limitations, restorations with SDR Plus composites offered clinical application efficiency with improved fracture toughness and favorable optical integration.

## 1. Introduction

Dental caries is a major global health burden, affecting more than 2 billion people worldwide. It is one of the leading causes of loss of tooth structure and the necessity for restorative procedures [1]. As carious lesions extend into dentin, the biomechanical strength of the tooth becomes increasingly compromised, necessitating restorative approaches that can replace lost tissue and restore functional performance. However, this goal is challenging because enamel and dentin have different structural, mechanical, and optical properties. Conventional restorative methods that rely on a single material often fail to replicate this natural heterogeneity accurately, which can affect long-term clinical outcomes.

Resin-based composites have become the preferred material for direct restorative procedures because of their attractive appearance, good adhesive ability, and minimally invasive application [2]. However, concerns about their long-term durability persist. Research has shown that conventional resin composites are vulnerable to polymerization shrinkage, contraction stress, wear, marginal breakdown, and fracture under functional loads [3,4]. Clinical-failure studies have revealed that secondary caries and fractures are major reasons for failure in posterior composite restorations [5]. These issues highlight the necessity for restorative methods that can enhance stress distribution and strengthen the structure, especially in large posterior cavities exposed to high occlusal forces.

Consequently, restorative dentistry has shifted increasingly toward biomimetic concepts that aim to reproduce the hierarchical structure and biomechanical behavior of natural dental tissues. Within this framework, dentin-replacement materials (DRMs) have been introduced as intermediate restorative layers placed beneath the superficial enamel-mimicking composite layer. These materials are intended to simulate dentin-like mechanical behavior, provide internal support, absorb functional stresses, and reduce stress concentration at the adhesive interface [6]. Such a bilayered restorative approach has been increasingly associated with improved biomechanical performance and enhanced restoration longevity.

From the perspective of materials science, the mechanical performance of DRMs is strongly influenced by the composition of the polymer matrix, filler distribution, fiber reinforcement, and the integrity of the filler–matrix interface. These factors directly affect polymerization behavior, resistance to crack propagation, stress dissipation, and the optical performance of layered restorations. Furthermore, differences in the refractive index (RI) between restorative layers may influence translucency and final color integration, thereby affecting the esthetic outcome of biomimetic restorations.

Among DRMs, bulk-fill composites, resin-modified glass ionomers (RMGIs), and short fiber-reinforced composites (SFRCs) have attracted considerable interest because of their distinct mechanical and clinical characteristics. Bulk-fill composites were developed to simplify restorative procedures through placement in increments of up to 4–5 mm while maintaining an adequate depth of cure and reduced polymerization stress [7]. These materials offer improved clinical efficiency; however, their ability to minimize contraction stress and maintain long-term marginal integrity in deep posterior restorations remains controversial [8]. Moreover, concerns regarding their wear resistance and mechanical stability under prolonged occlusal loading persist.

RMGIs represent another restorative option that combines fluoride release and chemical adhesion with improved mechanical performance compared with conventional glass ionomer cements [9]. Their relatively low elastic modulus may provide a stress-absorbing effect that could be beneficial beneath resin composite restorations. However, investigations have demonstrated that water sorption and hydrolytic degradation may adversely affect their long-term structural stability and color durability [10]. Moreover, variations in handling characteristics and moisture sensitivity during placement continue to present clinical challenges.

In contrast, SFRCs have emerged as promising DRMs for restoring structurally compromised posterior teeth. The incorporation of randomly oriented short glass fibers enhances fracture toughness through stress redistribution, crack deflection, and crack-stopping mechanisms [11]. Vallittu and colleagues established the reinforcing effect of fiber incorporation on composite restorations, highlighting the importance of fiber orientation and filler interaction in improving load-bearing capacity [12]. Despite their favorable mechanical behavior, SFRCs may present esthetic limitations because the optical properties of embedded fibers can influence translucency and the final color matching of layered restorations [13].

Despite growing interest in dentin-replacement strategies, researchers have predominantly investigated mechanical and optical properties independently. Fracture toughness is a prioritized property for characterizing resin composites, as it reflects the material’s resistance to crack propagation that leads to failure under functional load, making it highly relevant to the clinical failure modes of composite restorations [14,15]. Further, color matching remains essential for achieving clinically acceptable esthetic outcomes, particularly in biomimetic restorative systems whereby the optical interaction between restorative layers influences the final appearance [16]. Comparative studies evaluating the combined influence of different DRMs on fracture behavior and final color integration of layered restorations are scarce. Furthermore, clinically relevant factors such as application efficiency and handling characteristics continue to be underreported despite their direct influence on operator performance and clinical decision-making. To the best of the authors’ knowledge, comparative evaluation of different DRMs, particularly between bulk-fill composites, SFRCs, and RMGIs as DRMs, by simultaneously evaluating fracture toughness, color matching, and handling have not been examined within single standardized experimental framework. Therefore, this study was conducted to address this gap in the literature and aimed to evaluate the fracture toughness, color match, and application efficiency of resin composite restoration using different DRMs. The null hypothesis was that the DRM type would not markedly influence the fracture toughness, color integration, or application efficiency of layered resin composite restorations.

## 2. Materials and Methods

### 2.1. Study Design and Setting

**Figure 1:**
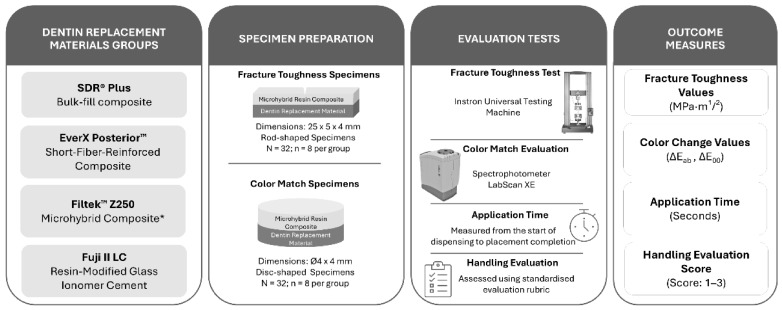
Study design (schematic). * represents control group.

This in vitro study evaluated four DRMs: bulk-fill composite (SDR^®^ Plus Bulk Fill Flowable, Dentsply Sirona, DE, USA); SFRC (EverX Posterior^™^, GC Corp., Tokyo, Japan); conventional composite, which served as control (Filtek^™^ Z250 Universal Restorative, 3M ESPE, St. Paul, MN, USA); and RMGI (Fuji II LC^®^ Capsule, Tokyo, Japan). The study design is illustrated in Figure 1. Specimens comprised the tested DRM layered with conventional resin composite material to simulate dental restoration. Two sets of specimens were prepared (N = 32 per set) of standardized dimensions to satisfy the testing requirements. A set of rod-shaped specimens was subjected to a fracture-toughness testing. A set of disc-shaped specimens was optically evaluated with an objective color analysis test. Evaluation of material handling was carried out by two independent assessors using a standardized rubric-based evaluation and scoring approach. Specimens were prepared and handling was evaluated in the clinical simulation laboratories at the College of Dentistry, King Saud bin Abdulaziz University for Health Sciences (Riyadh, Saudi Arabia). Physical and optical testing were done in the physical testing research laboratories at the College of Dentistry, King Saud University (Riyadh, Saudi Arabia).

### 2.2. Specimen Preparation

Specimens were prepared as layered resin composite restorations with four distinct DRMs: group A (SDR^®^ Plus, bulk-fill composite), group B (EverX Posterior™, SFRC), group C (Filtek™ Z250, conventional microhybrid composite), and group D (Fuji II LC, RMGI cement). Information on materials and group codes given for blinding are listed in Table 1. Sixty-four specimens were fabricated: 32 rod-shaped specimens for fracture-toughness testing and 32 disc-shaped specimens for color-match analysis (n = 8/group for each test). The sample size was determined based on similar in vitro studies with highly controlled experimental set-ups evaluating physical properties of resin composites and was found sufficient for detecting significant differences in the planned outcome measures [17,18]. All specimens were prepared by two co-authors of this article, who are dentists with professional Doctor of Dental Medicine degree (DMD) qualifications.

Specimens for fracture-toughness testing were prepared using a mold of 25 mm in length, 5 mm in width, and 4 mm in height with a single-edged notch of 2 mm in depth to facilitate controlled crack initiation, in accordance with the method described by Al-Angari et al. (2014) [19], which follows ISO 13586 [20]. Specimens used for color-match analysis were prepared using a mold of 4 mm in diameter and 4 mm in height. The thickness of applied materials in each specimen was carefully controlled using predefined reference points within the mold to ensure precise and consistent material thickness across all samples. Each material was applied with a uniform thickness of 2 mm for DRMs and 2 mm of the overlaying composite. For group A (SDR® Plus), the material was applied using the manufacturer-provided UniTip delivery system to ensure controlled and standardized placement. For group B (EverX Posterior™), the material was similarly dispensed using the UniTip delivery system to maintain consistency in application. For group C (Filtek™ Z250 Universal Restorative), the composite was placed incrementally and adapted into the mold. For group D (Fuji II LC), capsules were mixed mechanically for 10 s using an amalgamator (Softly, Acteon, France) operating at a speed of 4000 oscillations per minute (~66.7 Hz), then dispensed immediately into the mold according to manufacturer instructions. Each layer was light-cured for 20 s using a Demi Ultra LED curing unit (Kerr Corporation, Brea, CA, USA). For rod-shaped specimens, the beam of curing light was projected to three zones across the length of the specimen for 20 s in each zone to ensure optimal polymerization. Following application of DRMs, all specimens were overlayed with a microhybrid resin composite restorative material to replicate restoring of the dental enamel layer above the applied DRM in a restoration. Filtek™ Z250 was chosen for this purpose owing to its conventional nature, and it has been extensively studied and used as a benchmark in comparative studies [21]. The overlaying composite material was applied then covered with a Mylar strip and glass slab to obtain a flat surface, and light-cured for an additional 20 s using the same curing unit (Demi Ultra LED). Light-curing time was standardized using a timer to ensure consistent polymerization time. The adequate output intensity of the light-curing unit was verified using the associated radiometer prior to specimen preparation. Excess material was removed, and all specimens were finished and polished using a multi-step aluminum oxide polishing disc system (Sof-Lex™; 3M ESPE, Saint Paul, MN, USA) to standardize surface smoothness across all groups.

Each specimen within the group was labeled sequentially from 1 to 8 with a specific color and letter coding for groups. Labels were engraved on the side of rod-shaped specimens and on the base of disc-shaped specimens using a quarter carbide bur mounted on a low-speed handpiece for accurate specimen tracking. Each specimen was stored individually in a labeled plastic bag to ensure appropriate organization and traceability throughout experimental procedures.

### 2.3. Application Time and Handling Evaluation

Application time was recorded once per specimen in minutes and seconds for preparation of disc-shaped specimens because their application closely simulates clinical cavity restoration. A digital stopwatch (0.01 s precision) was used, with timing starting from DRM dispensing into the mold and ending after final light-curing of the overlying composite layer without including the pre-dispensing steps to control and standardize the test for all groups.

The handling of tested materials was evaluated by two independent assessors (F.A.A. and M.N.A.), co-authors of this study and qualified dentists with a DMD, under standardized in vitro conditions. To enable structured and consistent evaluation of the handling outcome measures investigated in this study, a rubric-based scoring system was developed. Before commencing the evaluation, the assessors reviewed the proposed evaluation criteria and scoring levels to ensure common understanding of criteria interpretation and scoring expectations. The outcomes of the review session were discussed among the investigators/assessors, followed by refinement of the scoring system through consensus, including reducing the scoring scale from a 5-point to 3-point Likert scale to improve discrimination across the scoring levels. The assessment criteria in the finalized scoring system (Table 2) include: viscosity and flow behavior; stickiness to instruments; ease of material placement into the mold; adaptation to cavity walls; surface smoothness before curing; and overall operator satisfaction. Each criterion was scored according to predefined criteria: 1 indicated “poor” performance; 2 indicated “good” performance; 3 indicated “excellent” performance. The scores obtained from both assessors were recorded and analyzed. Interoperator reliability was assessed using weighted Cohen’s kappa coefficient (κ).

### 2.4. Fracture-Toughness Test

Fracture toughness (*K_IC_*) was evaluated using a three-point bending test conducted on a universal testing machine (5900 series; Instron, Norwood, MA, USA) equipped with a 5-kN load cell. A centrally positioned loading indenter was applied at a constant crosshead speed of 0.5 mm/min. The maximum load value was recorded using Bluehill software (version 3.22.1373, Norwood, MA, USA). The fracture toughness K_IC_ (expressed in MPa·m^1^/^2^) was calculated as described in Al-Angari et al. (2014) [19], which was based on ISO 13586 [20] using the following formula:

$$K_{IC}\;=\;f\left(\frac{a}{w}\right)\left(\frac{F}{h\sqrt{w}}\right)$$

where a denotes the crack length in mm, w is the width of the specimen in mm, h is the height of the specimen in mm, w is the width of the specimen in mm, F represents the load in N, and f(a/w) is the fracture geometry factor computed using the following equation:

$$6\alpha ½\;[1.99\;-\;\alpha (1-\;\alpha )(2.15\;-\;3.93\alpha \;+\;2.7\alpha^{2})]\;/\;[(1+2\alpha )(1-\alpha )3/2]$$

### 2.5. Color Analysis

Color-match analysis was done using a calibrated spectrophotometer (LabScan XE; HunterLab, Reston, VA, USA). Due to the discrepancy between the specimen diameter (4 mm) and the spectrophotometer measurement aperture (5 mm), the base of specimens was embedded in a clear autopolymerizing resin using custom polyvinyl chloride housing molds. Reproducible positioning of specimens during embedding was achieved with the aid of a customized silicon putty index centering guide. Specimen embedding was done to prevent “edge loss” and light scattering by providing a continuous surface for the measurement port, thereby standardizing the background and peripheral interface for all groups. Embedded specimens were labeled then evaluated for measurement of color match. Color-match measurements were obtained at three standardized points on each specimen surface to account for potential surface variability and to improve measurement reliability. Measurements were taken at positions corresponding to 12 o’clock, 12:15, and 12:30 orientations. Recorded values were averaged to obtain a representative color value for each specimen. The color parameters (CIE L*a*b*) were determined using EasyMatchQC software (version 4.90, HunterLab, Reston, VA, USA). The mean CIE L*a*b* of Group C (Filtek Z250 resin composite) was established as the reference value. The color match for every individual specimen across all experimental groups, including individual specimens within Group C, was determined relative to the defined reference CIE L*a* b* value using ΔE*_ab_ and ΔE*_00_ metrics computed with the following equations:

$$\Delta E_{ab}^{*}\;=\;\sqrt{{\left({\;\Delta L}^{*}\right)}^{2}+{\left({\;\Delta a}^{*}\right)}^{2}+{\left(\;\Delta b^{*}\right)}^{2}}$$

where ΔE*ab represents the total color change, L represents the lightness, a represents green-red chromaticity coordinate, and b represents the blue-yellow chromaticity coordinate.

$$\Delta E_{00}^{*}=\sqrt{\left[{\left(\frac{\Delta L^{\prime }}{K_{L}\;S_{L}}\right)}^{2}+{\left(\frac{\Delta C^{\prime }}{K_{C}\;S_{C}}\right)}^{2}+{\left(\frac{\Delta H^{\prime }}{K_{H}\;S_{H}}\right)}^{2}+RT\left(\frac{\Delta C^{\prime }}{K_{C}\;S_{C}}\right)\left(\frac{\Delta H^{\prime }}{K_{H}\;S_{H}}\right)\right]}$$

where ΔE*_00_ represents the total color difference; ΔL, ΔC, and ΔH represent the differences in lightness, chroma, and hue, respectively; S_L_, S_C_, and S_H_ are the weighting functions for each component; K_L_, K_C_, and K_H_ are the parametric factors for the viewing conditions; and RT is the rotation function for chroma and hue interactions in the blue region.

### 2.6. Statistical Analyses

Descriptive statistics were calculated. Results are expressed as the mean ± standard deviation (SD). The normality of data distribution was assessed using the Shapiro–Wilk test prior to inferential analysis. One-way analysis of variance (ANOVA) followed by Tukey’s post hoc test was used to compare fracture toughness, color difference (ΔE), and application time between groups. Data for the application time are summarized as the mean ± SD and group comparisons were performed using ANOVA followed by Tukey’s post hoc test. Scores for handling evaluation are expressed as the mean ± SD and median (IQR), and analyzed using the Kruskal–Wallis test followed by post hoc pairwise comparisons. Interoperator reliability was assessed using weighted Cohen’s κ. Data were analyzed using Python software (version 3.10, VA, USA) utilizing the SciPy, Statsmodels, and Scikit-posthocs libraries. The level of significance was set at *p* < 0.05.

## 3. Results

### 3.1. Application Time

**Figure 2:**
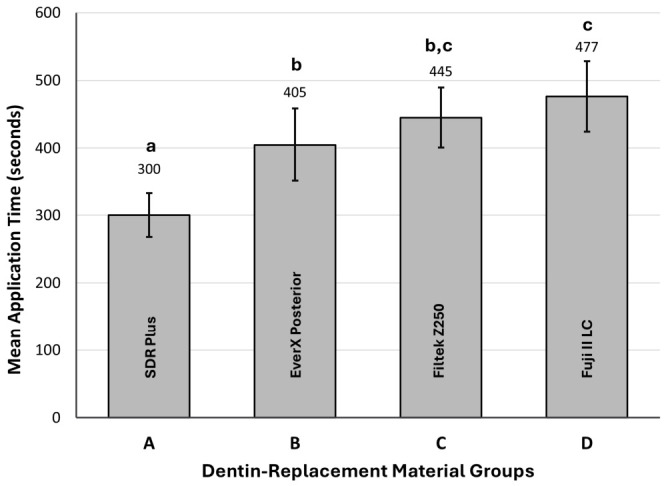
Comparison of application time across study groups. Error bars represent standard deviation. Groups with same letters are not significantly different (*p* < 0.05).

The data for application efficiency are presented in Figure 2. Group A demonstrated the shortest mean application time (300.25 ± 32.45 s), whereas group D required the longest application time (476.56 ± 52.07 s). Normality and homogeneity criteria were satisfied. One-way ANOVA indicated a highly significant difference in application time among experimental groups (F(3, 28) = 21.98, *p* < 0.001, η^2^ = 0.702).

Post hoc comparisons via Tukey’s test revealed that group A required significantly less time than groups B, C, and D (all *p* < 0.001). Group D required a significantly longer application time compared with that for group B (*p* = 0.022). Significant differences were not detected between group B and group C (*p* = 0.322) or between group C and group D (*p* = 0.539).

### 3.2. Handling Scores

**Figure 3:**
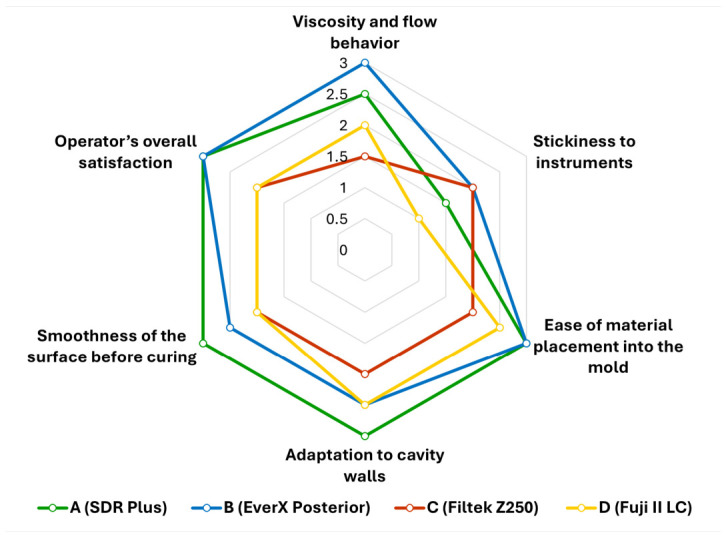
Averaged scores of handling criteria for tested materials in groups in a radar chart.

The average scores of handling criteria for each group are depicted in Figure 3. The overall mean and median handling scores for groups are listed in Table 3. The total mean handling score ranged from 1.92 ± 0.20 in group C to 2.67 ± 0.61 in group A. Descriptive distribution trends indicated that group C received the least overall handling score, while groups A and B consistently displayed the highest score. The overall weighted Cohen’s κ was 0.53, establishing a moderate baseline agreement among the evaluators.

### 3.3. Fracture Toughness

**Figure 4:**
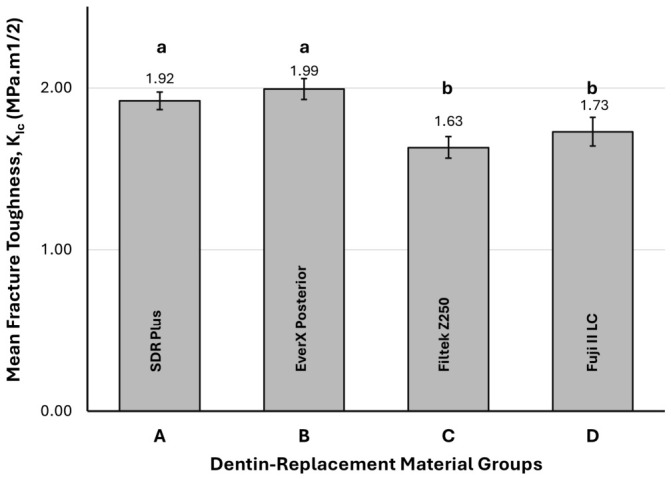
Comparison of fracture toughness across study groups. Error bars represent standard deviation. Groups with same letters are not significantly different (*p* < 0.05).

The distinct performance tiers are graphically depicted in Figure 4. Group C demonstrated the lowest mean fracture toughness (1.63 ± 0.06), whereas group B exhibited the highest mean fracture toughness (1.99 ± 0.06). One-way ANOVA revealed a highly significant difference in fracture toughness across the tested groups (F(3, 28) = 39.94, *p* < 0.001, η^2^ = 0.811). All parametric assumptions required for the analysis of variance were fully satisfied. Post hoc multiple comparisons using Tukey’s test revealed that group A and group B exhibited significantly greater fracture toughness compared with those in groups C and D (all *p* < 0.001). Conversely, a significant difference was not detected between group A and group B (*p* = 0.230), nor between group C and group D (*p* = 0.062).

### 3.4. Color Difference (ΔEab and E00)

**Figure 5:**
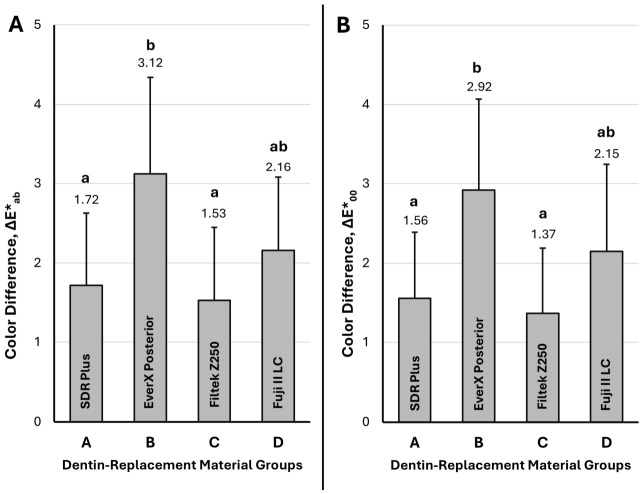
Comparison of color difference across study groups. (**A**) ΔE*ab values and (**B**) E00 values. Error bars represent standard deviation. Groups with same letters are not significantly different (*p* < 0.05).

**Figure 6:**
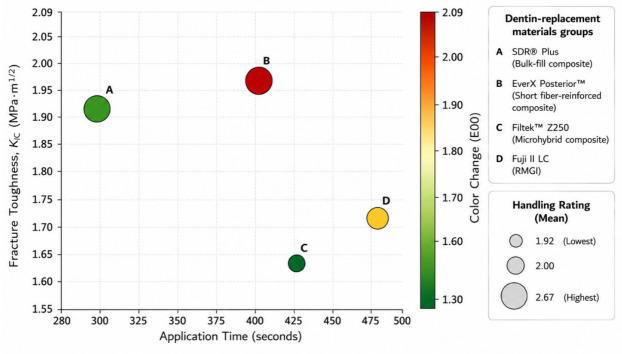
Graphical summary illustrating the performance of the tested dentin-replacement materials.

This statistical distribution and pattern of color variation are illustrated graphically in Figure 5. The mean ΔE*ab ranged from 1.53 ± 0.92 in group C to 3.12 ± 1.22 in group B. The E00 ranged from 1.37 ± 0.82 in group C to 2.92 ± 1.15 in group B. Before inferential statistical analyses, all datasets were confirmed to meet the assumptions of normality (Shapiro–Wilk test, *p* > 0.05) and homogeneity of variance (Levene’s test, *p* > 0.05), justifying the application of one-way ANOVA. A significant difference in ΔE*ab was detected among experimental groups (F(3, 28) = 4.04, *p* = 0.017, η^2^ = 0.302). Subsequent post hoc pairwise comparisons utilizing Tukey’s test revealed that group B exhibited significantly higher ΔE*ab values compared with those in group A (*p* = 0.042) and group C (*p* = 0.018). Significant differences were not observed between any other pairs of groups (*p* > 0.05). Evaluation of E00 values demonstrated a significant difference across groups (F(3, 28) = 4.03, *p* = 0.017, η^2^ = 0.302). Post hoc analysis confirmed that group B had considerably higher E00 values than those in group A (*p* = 0.045) or group C (*p* = 0.019). In alignment with the findings for ΔE*ab, significant differences were not detected between the remaining experimental groups (Figure 5B). A comprehensive multidimensional mapping of these color variations alongside fracture toughness, application time, and handling ratings across all tested groups is visually summarized in Figure 6.

## 4. Discussion

The primary goal of our study was to thoroughly assess the performance of resin composite restorations supported by different DRMs. The latter were a bulk-fill composite, an SFRC, a conventional composite, and an RMGI. We examined their mechanical fracture toughness, esthetic color matching, and handling efficiency. Overall, the results showed notable performance patterns among the tested restorative strategies. The restorations supported by the SFRC and bulk-fill composite exhibited superior mechanical strength; however, the SFRC faced challenges in esthetic integration. In contrast, the restorations supported by the RMGI as a DRM demonstrated lower mechanical resistance, but offered an acceptable esthetic integration. Collectively, the data indicated that the bulk-fill composite provided the most balanced profile, effectively bridging the gap between high fracture resistance, rapid application, and acceptable color matching.

The distinct physical behaviors observed among groups were chemically governed by their monomer systems and filler designs. The capacity of the bulk-fill composite to be placed in efficient 4 mm increments without suffering from high polymerization shrinkage stress was driven by its modified urethane dimethacrylate (UDMA) backbone, which incorporates modulators of chemical polymerization [22]. Micro-gel contraction during free-radical polymerization creates considerable internal stress in traditional high-molecular-weight resins such as Bis-GMA [23]. The integration of polymerization modulators allows the polymer network of this bulk-fill material to reorganize dynamically during light exposure, delaying the gel point without compromising the final degree of conversion [23,24]. The high mechanical resistance of the SFRC stems from its semi-interpenetrating polymer network (semi-IPN) matrix, which consists of crosslinked dimethacrylates intertwined with linear polymer chains [17]. This design forms a highly stable structural interface with its randomly oriented E-glass fiber filler sub-base [25]. This unique configuration enables effective load distribution away from the weaker resin matrix and directly onto high-tensile glass fibers, a trait that traditional particulate-filled resin systems lack [25]. Conventional composites rely on the standard crosslinking combinations of Bis–GMA, UDMA, and Bis–EMA [23]. This strategy yields a dense, stable network; however, it lacks specialized pathways of chemical-stress relaxation, which explains the lowest fracture resistance observed in the current study. Similarly, RMGIs utilize a dual-cure mechanism in which a classic acid–base glass ionomer gelation reaction proceeds alongside a light-initiated polymerization of hydrophilic resins [9]. This competing dual-setting pathway frequently introduces microscopic air voids and spatial inhomogeneities into the fully set matrix, thereby compromising its baseline predictability [26].

Fracture toughness (K_IC_) provides a direct measure of the intrinsic capacity of a material to resist catastrophic propagation of flaws under occlusal forces. In the present study, bulk-fill composite and SFRC groups exhibited statistically significant higher fracture resistance compared with those in the conventional composite and RMGI groups. This mechanical distinction is clinically vital because restorative materials presenting low fracture resistance are strongly correlated with increased long-term clinical failure, marginal degradation, and enamel cracking under load [14,27]. On the other hand, composites with high fracture toughness possess high resistance to wear [28], mitigating a major factor that compromises resin restoration longevity under physiological or pathological occlusal conditions. Of the tested restorations, the composites supported by the EverX posterior yielded the highest fracture toughness. This outcome is consistent with the superior structural resilience reported by Bijelic-Donova et al. (2016) [17] and the resistance to extreme fatigue loading and highly limited crack propensity reported by Soares et al. (2018) [29]. The structural mechanics of a fiber-reinforced system rely on classic energy-dissipation behaviors: fiber bridging and fiber pullout [25]. If a microcrack begins to propagate through the brittle matrix under load, the randomly oriented E-glass fibers cross the crack pathway, absorbing localized energy and redirecting stress paths horizontally [17]. This macromechanical shielding behavior closely mimics the structural role of collagen networks within natural dentin [30]. SDR Plus secured the second highest fracture toughness in our study with no significant difference from the EverX posterior results, closely mirroring the robust mechanical stability observed by Kose et al. (2025) when using this bulk-fill flowable as a supportive base layer [31]. This performance hierarchy is further validated by the physical characterizations of Attik et al. (2022), confirming that while fiber matrix integration sets the absolute ceiling for fracture resistance, the specialized, deep-curing chemistry of the SDR resin matrix still delivers an elevated threshold of core toughness that easily outclasses traditional materials [32]. Conversely, the lower fracture toughness observed in restorations supported by a conventional composite or RMGI is tied to their intrinsic limitations in the matrix. A conventional composite suffers from high monomer shrinkage and absence of stress relaxation pathways [22], but RMGI lack internal reinforcing skeletons combined with porosities in its structural matrix, which promote rapid crack propagation before the polyacid matrix can achieve full chemical maturation [26]. The inherent compositional properties of the conventional resin composite and RMGI make them a less suitable option as a DRM if resistance to stress is a primary consideration.

Achieving seamless blending between a tooth-colored restorative material and natural tooth structure/tissue requires a close replication of light scattering, absorption, and reflection, that are perceived as color [33]. Clinically, color is identified in a standardized shade scale that accounts for the hue, chroma, and value dimensions and is referred to as ‘shade’. The shade selection for tooth-colored restorative materials relies on matching the desired resultant shade of the outermost enamel layer shade with the underlying dentin layer shade. This was respected in our study as all tested DRMs were overlayed with a standardized resin composite restorative material intended to restore the enamel layer while serving as a substrate for assessing how the tested DRMs interact with it to yield the final color of the restoration. The type of the overlaying resin composite was carefully chosen to represent a well-known material a with large body of evidence, that allows results comparison with other studies. Further, the conventional nature of the overlaying resin composite material with the universal shade (A2), represented by Filtek Z250 resin composite restorative material, served as a clinically relevant reference for all color difference calculations. As Filtek Z250 resin composite is routinely used to restore both enamel and dentin, unlike the other tested materials that serve solely as DRMs, Group C exhibited a standard reference control for color-match analysis in the current study. Use of the ΔE_ab_ and ΔE_00_ formulas in our study provided a perceptually accurate assessment because it integrates visual corrections for human-eye sensitivity regarding chroma and hue changes [34]. The color analysis revealed that the SFRC exhibited statistically significant higher color variations compared with those of the other composite groups. This occurred because its highly translucent glass fibers created an optical window that allowed the deep darker background to show through [16]. This finding aligns with established clinical protocols noting that highly translucent dentin-replacement bases necessitate an overlying 2 mm surface capping layer of conventional composite to completely mask underlying optical boundaries and achieve ideal esthetic harmony [35]. The bulk-fill composite demonstrated an excellent color match, proving that it did not face the esthetic limitations often seen in advanced core-reinforcement materials. This ability to optically blend into the adjacent overlaying composite relies on minimizing the RI mismatch between structural inorganic fillers and the surrounding resin matrix [33]. The optimized UDMA–barium glass filler interface in a bulk-fill composite minimizes this RI variation, enabling a smooth esthetic transition.

Handling characteristics of restorative materials are closely linked to their rheological profile and impact the operator’s ability to manipulate the material with precision, ease, and efficiency. Handling properties of resin composites are extensively studied by laboratory objective methods such as stickiness, slump resistance, viscosity and porosity [36]. These highly controlled methods provide valuable specific characterization output results associated with placement technique sensitivity and allow differentiation of the tested materials in this respect [36]. As the material’s handling experience reflects the combined outcomes of the material’s handling properties and the interplay between the operator, the dental material and the clinical procedure, the objective material properties should be complemented with handling characteristics assessed by the operator in a clinical/simulated clinical environment. Earlier dentist-perceived handling evaluations of resin-based restorative materials have yielded valuable information and demonstrated that clinical assessors can perceive handling characteristics such as ease of placement, adaptation and manipulation [37]. While clinician-reported handling assessment exhibited a dimension of material evaluation that is not fully perceived from the laboratory objective testing alone, its use presents inherent challenges. Variabilities in clinical procedures and management complexities cannot be isolated from the intended materials’ handling characteristics [38]. Additionally, the acknowledged variabilities related to assessor’s background training and experience account for the differences in evaluating the handling of a material. Despite these challenges, operators’ perceived handling evaluations continue to add clinically relevant insights to objective assessment with improved controlled factors implemented in simulated clinical environment. As there were no unified tests for handling characteristics, the current study follows previous researchers efforts in designing and implementing the handling characteristics evaluation approach [39]. We report the first attempt of handling evaluation for the tested DRMs perceived by the end-user, the dentist, in a standardized simulated clinic setup. In this evaluation, restorations with a bulk-fill composite demonstrated the highest clinical efficiency, requiring substantially less application time than the other restorative strategies. This clinical efficiency is a direct result of the low-viscosity, self-leveling properties of the modified UDMA monomer, which allows it to adapt intimately into complex cavity line angles without requiring extensive manual manipulation [22]. Recent developments in bulk-fill restorative systems have enabled their use as full-contour restorations without an overlaying composite capping layer in selected cases [40]. Although this approach may further simplify the restorative workflow and further reduce application time, the present study adopted a standardized layered restoration protocol to ensure a fair comparison among the tested dentin-replacement materials. The application of viscous EverX posterior, Filtek Z250, and Fuji II LC required significantly more time by an average of 100 s to 170 s. In contrast to our findings, Roulet et al. (2013) did not find effects of materials’ viscosity on the application speed [39]. The combination of high fracture resistance, reduced application time, and stable color profiles observed with restoration supported by the bulk-fill composite highlighted its versatility for deep, posterior restorations [41].

Our study provides valuable comparative data on the mechanical, esthetic, and handling aspects of various DRMs, but main limitations should be acknowledged and considered when interpreting the present findings. First, as an in vitro study, the experimental conditions cannot fully replicate the complex and dynamic environment of the oral cavity. The specimens were subjected to static three-point bending tests, which do not account for the cyclic fatigue, thermal fluctuations, and pH variations that restorations typically encounter in vivo. Future research should incorporate aging protocols, such as thermocycling and mechanical load cycling, to better predict the long-term clinical durability of these layered systems. Second, use of standardized rod and disc geometries, while necessary for accurate measurements of fracture toughness and color match, differ from the complex anatomy of tooth cavities. The flat surfaces and uniform thicknesses used in our study do not account for the C-factor and polymerization shrinkage stress generated in constricted cavity preparations. Clinical or ex vivo studies using extracted teeth and varied cavity designs (e.g., compound and complex class-II cavities) are necessary to validate these findings in more realistic anatomical scenarios. Third, the color-match analysis was conducted against a standardized black background to isolate material properties. In a clinical setting, the final esthetic outcome is heavily influenced by the background color of the remaining natural tooth structure (the “chameleon effect”). Investigating the tested bilayered systems over varying shades of natural dentin to determine the masking ability and esthetic integration of the DRM more accurately would be worthwhile. Another limitation concerns the subjective assessment of handling characteristics. Although a standardized evaluation rubric was used and the assessors received similar training, the inter-rater agreement reached a moderate level, indicating room for improvement in scoring consistency. Future studies should further validate the rubric and include additional calibrated evaluators to enhance inter- and intra-rater reliability. Despite these limitations, a key strength of this research is its multidimensional approach. By evaluating mechanical fracture toughness, perceptual color differences, and operator-dependent handling characteristics concurrently, our study offers a more holistic view of material performance than traditional single-parameter evaluations. Furthermore, inclusion of the application time provides a practical, clinic-centric metric that relates materials science directly to chairside efficiency.

## 5. Conclusions

Within the limitations of this in vitro study, EverX Posterior and SDR Plus showed promising mechanical performance as dentin-replacement materials. Specifically, SDR Plus exhibited a balanced performance profile, characterized by improved fracture toughness, an acceptable color match, and a quick application under laboratory conditions. To build on these laboratory results, further long-term clinical trials and in vivo studies are recommended to fully assess the durability, performance, and esthetic integration of these layered restorative systems in the dynamic oral environment.

## Figures and Tables

**Table 1:** The dentin-replacement materials used in this study.

Group	Product (Shade)/Type	Manufacturer	Resin Composition	Filler Characteristics
A	SDR^®^ Plus Bulk Fill (A2)/Bulk-fill composite	Dentsply Sirona, Milford, DE, USA	Modified UDMA resin with barium glass fillers	Composition: Barium alumino-fluoro-borosilicate glass, strontium alumino-fluoro-silicate glass, fumed silica, ytterbium fluoride Loading: 70.5 wt.%/47.4 vol.% Particle size: 4.2 µm (mean)
B	EverX Posterior™ (Universal shade)/Short fiber-reinforced composite (SFRC)	GC Corporation, Tokyo, Japan	Bis-GMA, PMMA, TEGDMA + E-glass fibers	Composition: E-glass fibers, barium glass fillers Loading: 74.2 wt.%/53.6 vol.% Fibers size: 800 × 17 µm; glass particle size: 0.02–0.7 µm
C	Filtek™ Z250 Universal Restorative (A2)/Microhybrid composite	3M ESPE, St. Paul, MN, USA	Bis-GMA, UDMA, Bis-EMA with zirconia/silica fillers	Composition: Zirconia/silica fillers Loading: 82 wt.%/60 vol.% Particle size: 0.01–3.5 µm
D	Fuji II LC Capsule (A2)/Resin-modified glass ionomer (RMGIC)	GC Corporation, Tokyo, Japan	polyacrylic acid + HEMA	Composition: Fluoro-aluminosilicate glass Loading: 76.2 wt.%/55 vol.% Particle size: ≈5.9 µm

UDMA, urethane dimethacrylate; TEGDMA, triethylene glycol dimethacrylate; Bis-GMA, bisphenol A-glycidyl methacrylate; Bis-EMA, ethoxylated bisphenol A dimethacrylate; PMMA, polymethyl methacrylate; HEMA, 2-hydroxyethyl methacrylate; SFRC, short fiber-reinforced composite; RMGIC, resin-modified glass ionomer cement.

**Table 2:** Criteria for evaluating handling and the scoring system rubric.

Criteria	Score Description		
	Excellent (3)	Good (2)	Poor (1)
1. Viscosity and flow	Material flows easily without excessive pressure; readily adapts to cavity walls under gravity or light instrumentation	Material has moderate viscosity; manageable flow with slight adaptation assistance	Material is overly viscous or stiff; does not flow easily into the cavity; requires forceful placement
2. Stickiness to instruments	Material does not stick to instruments; allows clean, precise manipulation	Material shows some stickiness but can be managed with careful placement	Material sticks excessively to instruments; hard to handle; stringy or pulls out of the cavity
3. Ease of placement	Placement is smooth and accurate; minimal effort needed for adaptation or positioning	Generally easy to place; may require minor corrections or positioning	Difficult to place or position material correctly; tends to displace or requires repeated adjustment
4. Adaptation to cavity walls	Material closely conforms to all cavity walls and surfaces without manipulation	Adaptation is acceptable but may require moderate condensation or manipulation	Gaps or voids visible; does not adapt well to the cavity, especially in internal angles
5. Surface smoothness after placement	Surface is even and smooth immediately after placement without adjustment	Minor surface roughness; smooths out with light adjustment	Irregular surface; rough or lumpy texture visible even before curing
6. Operator satisfaction (overall handling experience)	Operator was fully satisfied with the handling experience; smooth and predictable	Operator was moderately satisfied; some issues noted but acceptable overall	Operator was dissatisfied with handling; found it difficult or frustrating to use

**Table 3:** Overall handling scores across groups.

Variable	Group A	Group B	Group C	Group D
Handling score, mean ± SD	2.67 ± 0.61	2.67 ± 0.41	1.92 ± 0.20	2.00 ± 0.55
Median (IQR)	3 (2.63–3.00)	2.75 (2.50–3.00)	2 (2–2)	2 (2–2.38)

## Data Availability

The raw data supporting the conclusions of this article will be made available by the authors on request.

## Abbreviations

The following abbreviations are used in this manuscript:

ANOVA	Analysis of variance
CIE	International Commission on Illumination
DMD	Doctor of Dental Medicine
DRM	Dentin-replacement materials
RI	Refractive index
RMGI	Resin-modified glass ionomers
SD	Standard deviation
SFRC	Short fiber-reinforced composite
